# The effect of body armour and load carriage on respiratory function and exercise

**DOI:** 10.1186/2046-7648-4-S1-A84

**Published:** 2015-09-14

**Authors:** Nicola Armstrong, Amanda Ward, Gilbert Chanza, Mitch Lomax, Michael J Tipton, James R House

**Affiliations:** 1 Dstl, Human Systems Group, Porton Down, Salisbury, UK; 2 Extreme Environments Laboratory, Department of Sport and Exercise Science, University of Portsmouth, Portsmouth, UK

## Introduction

Wearing body armour (BA) causes a restrictive respiratory defect, which is caused by its increased mass and restriction of chest expansion [1,2]. The evidence suggests that this respiratory impairment is sufficient to reduce maximal exercise capacity and may result in the early onset of fatigue [3]. This study tested the hypothesis that UK military BA and load carriage would impair respiratory function at rest and during military patrolling tasks.

## Methods

24 male military participants completed a laboratory test five times wearing no BA, BA alone (total mass 15 kg), BA + 15 kg (30 kg), BA + 25 kg (40 kg) or BA + 35 kg (50 kg). The laboratory test involved measurements of pulmonary function at rest (*e.g*. flow volume loops) and during a continuous treadmill test (*e.g*. tidal flow volume loop measurement during exercise). The speed and incline (%) of the treadmill was increased every ten minutes to represent the following military tasks; a cautious patrol (light exercise: 3 km.h^-1^, 0 %), low threat patrol (moderate exercise: 4 km.h^-1^, 3 %), forced march (heavy exercise: 5 km.h^-1^, 4 %) and contact situation (very heavy exercise: 6 km.h^-1^, 5 %). Respiratory muscle pressures (RMP) were also measured pre and post exercise.

## Results

**Figure 1:**
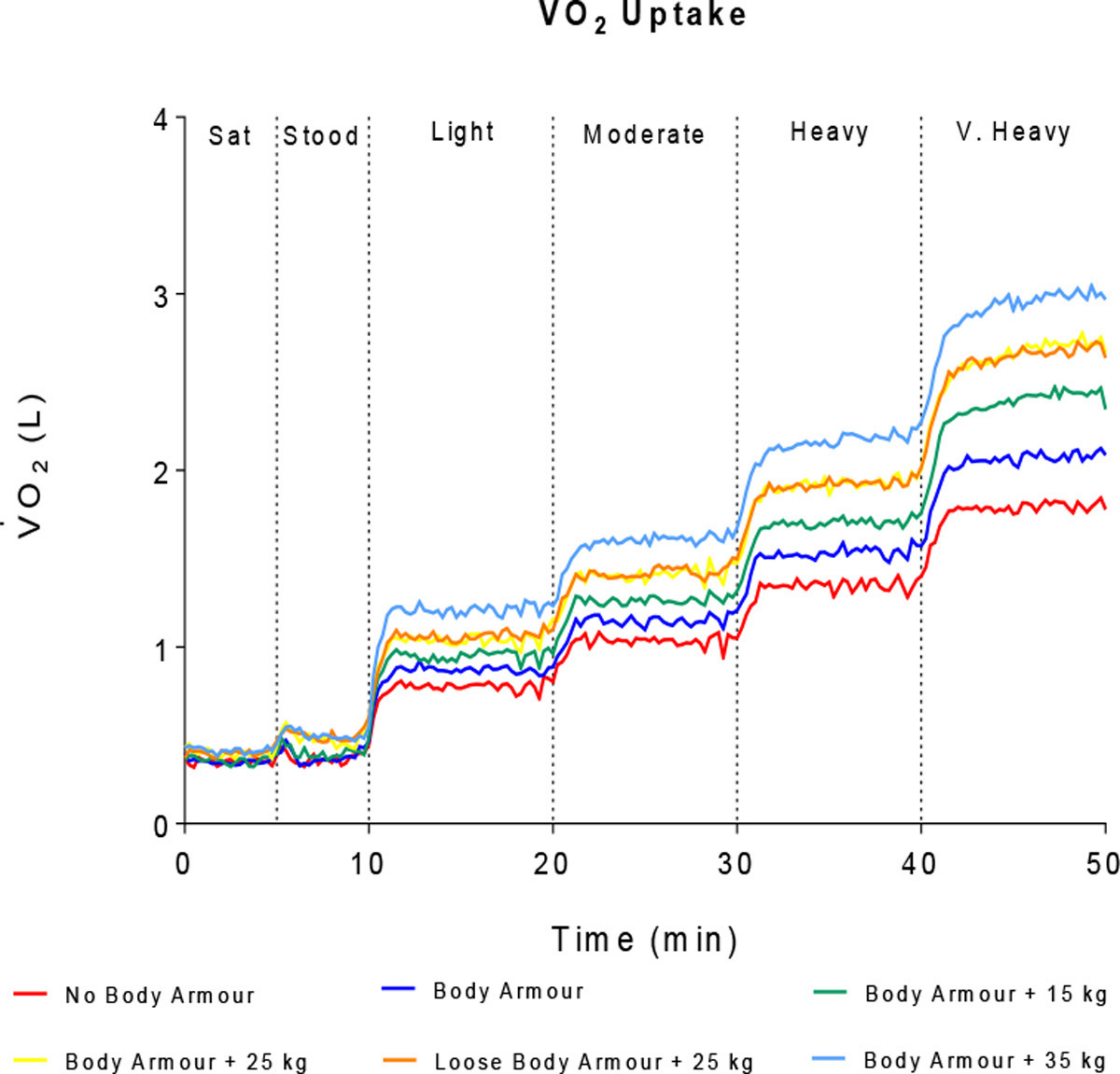


BA caused a mild (6 % to 8 %) restrictive respiratory impairment (a reduction in FVC and FEV_1 _without a reduction in FEV_1_/FVC). This restriction increased to 15 % with the addition of 35 kg. A reduction in RMP was observed in all conditions immediately post exercise (11 % to 18 % for inspiratory muscles and 16 % to 23 % for expiratory muscles). At five minutes post exercise, RMP returned to baseline levels when no BA was worn and also when BA was worn without additional load. Respiratory limitation was observed during very heavy exercise in the loaded conditions (where exercise tidal flows meet the maximum flow volume loop envelope). Energy expenditure was defined for five loads and four exercise intensities (see Figure 1). At each exercise intensity, increases in ventilation caused by wearing BA and load were explained by increases in breathing frequency.

## Discussion

This study has quantified the respiratory burden associated with carrying BA with loads up to 50 kg. A restrictive respiratory impairment was observed with BA and load carriage. Reductions in RMP are indicative of respiratory muscle fatigue and suggest that longer periods of recovery would be required between tasks when BA is worn with load.

## Conclusion

UK military soldiers may experience respiratory limitation during patrolling tasks. This may reduce exercise capacity and lead to the early onset of fatigue [3]. These findings have implications for military task performance *e.g*. marksmanship and fire and movement, where stable breathing patterns and quick recovery times are essential.
